# Review of the Role of TRAF7 in Brain Endothelial Integrity and Cerebrovascular Aging

**DOI:** 10.3390/life15081280

**Published:** 2025-08-12

**Authors:** Jennifer Ihuoma, Sherwin Tavakol, Sharon Negri, Cade Ballard, Khanh Phan, Albert Orock, Zeke Reyff, Madison Milan, Eva Troyano-Rodriguez, Rakesh Rudraboina, Anna Csiszar, Anthony C. Johnson, Ian F. Dunn, Stefano Tarantini

**Affiliations:** 1 Vascular Cognitive Impairment, Neurodegeneration and Healthy Brain Aging Program, Department of Neurosurgery, University of Oklahoma Health Sciences Center, Oklahoma City, OK 73104, USA; jennifer-ihuoma@ouhsc.edu (J.I.); sherwin-tavakol@ouhsc.edu (S.T.); sharon-negri@ouhsc.edu (S.N.); cade-ballard@ouhsc.edu (C.B.); zeke-reyff@ouhsc.edu (Z.R.); madison-milan@ouhsc.edu (M.M.); eva-troyanorodriguez@ouhsc.edu (E.T.-R.); rakesh-rudraboina@ouhsc.edu (R.R.); anna-csiszar@ouhsc.edu (A.C.); 2 Oklahoma Center for Geroscience and Healthy Brain Aging, University of Oklahoma Health Sciences Center, Oklahoma City, OK 73104, USA; 3 Department of Neurosurgery, University of Oklahoma Health Sciences Center, Oklahoma City, OK 73104, USA; khanh-phan@ouhsc.edu (K.P.); albert-orock@ouhsc.edu (A.O.); anthony-johnson@ouhsc.edu (A.C.J.); 4 Department of Health Promotion Sciences, College of Public Health, University of Oklahoma Health Sciences Center, Oklahoma City, OK 73104, USA; 5 The Peggy and Charles Stephenson Cancer Center, University of Oklahoma Health Sciences Center, Oklahoma City, OK 73104, USA

**Keywords:** vascular fragility, cerebrovascular health, cognitive dementia, aging, TRAF7 protein, tumor necrosis factor, endothelial cell, MAPK, MEK5, ERK5

## Abstract

Tumor necrosis factor (TNF) receptor-associated factor 7 (TRAF7) is a signal transducer in the TNF receptor superfamily. TRAF7 is unique among its superfamily in that it does not contain a TRAF-C domain but does contain WD-40 domains. TRAF7 interacts with mitogen-activated protein kinases (MAPK), which are known regulators of inflammation and shear stress response. Notably, these molecular interactions have profound implications for the function of brain endothelial cells (ECs), which are pivotal for sustaining the integrity of the blood–brain barrier (BBB), orchestrating neurovascular coupling (NVC), and modulating the vascular architecture. By directly influencing MAPK signaling pathways, particularly the shear stress-responsive MAPK kinase kinase 3 (MEKK3)–MAPK kinase 5 (MEK5)–extracellular-regulated protein kinase 5 (ERK5) cascade, TRAF7 contributes to vascular homeostasis, as exemplified by its role in phosphorylating ERK5. Such molecular events underpin the capacity of brain ECs to regulate substance exchange, adjust blood flow in response to neural activity, and maintain efficient cerebral perfusion, all of which are essential for preserving brain health and cognitive performance. By synthesizing the current evidence regarding TRAF7’s molecular functions and its impact on brain endothelial integrity, cerebrovascular aging, and exploring implications for therapeutic strategies targeting vascular dysfunction in the aging brain, this review fills a crucial gap in the literature. Given the limited number of original studies directly addressing these contexts, the review will integrate broader insights from related literature to provide a foundational overview for future research in this developing field. The culmination of this literature will provide a rationale for the development of novel TRAF7-targeted therapies to restore vascular integrity in the context of aging, which could maintain cognitive health. Although TRAF7 has been implicated in regulating endothelial permeability during inflammation, its precise functions in brain ECs and the subsequent effects on cerebrovascular structure and cognitive function remain to be fully elucidated.

## 1. Introduction

Aging is a complex biological process that is associated with an increase in physiological and structural alterations due to the body’s decreased ability to respond to cellular stressors [1,2]. These alterations in the brain increase the risk of cognitive decline and the development of neurodegenerative diseases in the elderly [2]. It is well recognized that the brain relies on intracellular energy substrates to support neuronal metabolism, with oxygen and essential nutrients delivered via the cerebrovascular networks [3,4,5,6,7]. This network is also involved in the removal of cellular waste from the brain [8]. However, aging affects cerebrovascular health through vascular function alterations, long-term inflammation, elevated oxidative stress, reduced nitric oxide (NO) bioavailability, and hormonal changes [8] that promote the development of age-related vascular cognitive impairment (VCID) [9,10]. These factors lead to reduced cerebral blood flow (hypoperfusion), inefficient neuronal processing, diminished cerebrovascular reactivity, and impaired neurovascular coupling (NVC) [11,12,13,14,15]. Consequently, the aging cerebrovasculature makes the brain more vulnerable to cognitive deficits, including vascular cognitive impairment, dementia, delirium, and amnesia [16,17,18,19,20,21]. A comprehensive understanding of the molecular mechanisms underlying cerebrovascular aging is essential for developing effective therapeutic strategies to preserve brain health and cognitive function in the elderly.

Endothelial cells (ECs) are a critical component of the vascular system, forming the inner lining of blood vessels [5]. In the brain, vascular endothelial cells play a pivotal role in maintaining vascular health as they are integral components of the BBB [22,23,24,25] and the neurovascular unit, a functional ensemble of endothelial cells, glia, neurons, and pericytes that coordinates cerebral blood flow and maintains brain homeostasis [5,26,27,28,29,30,31,32,33,34]. Endothelial cells tightly regulate the transport between the brain and the periphery, protecting neural tissue from harmful substances, pathogens, and fluctuations in blood composition [26,29,35,36,37]. They are also crucial in NVC as they help regulate cerebral blood flow (CBF) in response to regional increases in cellular demand during neural activity [26,35,36]. In addition, endothelial cells influence inflammatory responses, angiogenesis, arterial remodeling, and the production of vasoactive substances, such as NO, endothelium-derived hyperpolarizing factor, eicosanoids, and endothelin, which are vital for maintaining adequate cerebral perfusion [5,36,38,39]. Consequently, endothelial cellular dysfunction can compromise the integrity of the BBB [23] and disrupt NVC, ultimately leading to cognitive decline [9,23,26,33,34,40,41].

**Figure 1:**
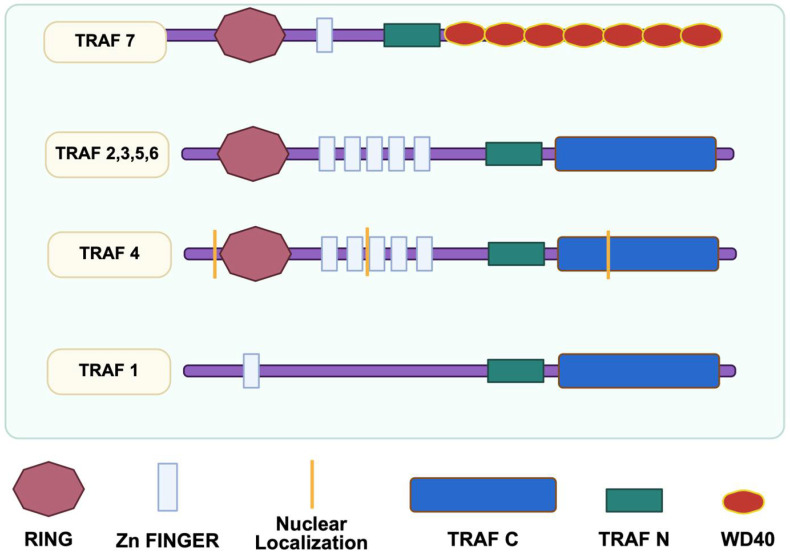
**TRAF protein structure.** TRAF proteins have a distinctive modular structure which enables them to act as adapter molecules for cell-surface receptors and regulate a variety of cellular responses. TRAF7 has seven WD40 repetitions at the C-terminus, unlike the other six TRAF proteins which have the TRAF-C domain at the C-terminus. The WD40 repetitions are nucleic acid or protein interaction interfaces, which allow TRAF7 to interact with certain kinases and receptors to affect stress and inflammatory responses [53].

Tumor necrosis factor (TNF) receptor-associated factors (TRAFs) are key mediators that link tumor necrosis factor receptors (TNFRs) to signaling cascades regulating the cellular effects of TNF family ligands. TRAF7, the most recently identified member of this family, plays a role in endothelial cell integrity, BBB permeability, promotion of neurovascular coupling, and maintenance of the vascular architecture. TRAF7 is unique because it lacks the typical TRAF-C domain; instead, it possesses seven tandem repeats of ~40 amino acids ending in tryptophan-aspartic acid (W-D) dipeptide (WD40 domain) at its carboxy terminus (C domain) (Figure 1) [42,43,44,45]. These WD40 repeats enable TRAF7 to interact synergistically with multiple factors, including mitogen-activated protein kinases (MAPKs), to regulate various transcription factors [45,46,47,48,49]. In endothelial cells, TRAF7 binds the carboxy terminus of roundabout guidance receptor 4 (Robo4), effectively reducing hyperpermeability during inflammatory responses [50,51]. Moreover, recent studies underscore the critical role of TRAF7 in the MAPK kinase kinase 3 (MEKK3)–MAPK kinase 5 (MEK5)–extracellular-regulated protein kinase 5 (ERK5) signaling pathway, which is essential for maintaining endothelial function in response to blood flow and shear stress, and thus preserving blood vessel integrity [48,52]. While there have been a number of recent reports that aim to elucidate TRAF7’s unique role in vascular function, the literature lacks a consolidating review that synthesizes the most up-to-date discoveries and highlights critical knowledge gaps that could serve as launching pads for future scientific inquiry. In this review, we examine the involvement of TRAF7 in modulating endothelial hyperpermeability, inflammatory responses, and shear stress signaling, highlighting its potential impact on cerebrovascular health during aging.

## 2. TRAF7 and Endothelial Function

### 2.1. Overview of the TRAF7 Structure and Signaling Pathways

Recent evidence indicates that TRAF7, the newest member of the TRAF family, plays a critical role in regulating endothelial cell function. TRAF7 was first characterized in 2004 in a study by Xu et al. [45], which highlighted its unique structure and function compared to other TRAF proteins. TRAF proteins are known to have a distinctive modular structure, which enables them to act as adapter molecules for cell-surface receptors and regulate a variety of cellular responses. TRAF7 has seven WD40 repetitions at the carboxy terminus [42,43,44,45] unlike other TRAF (1–6) proteins, which have the TRAF-C domain (Figure 1) [42,43,45,46,47]. WD40 repetitions are typically nucleic acid or protein interaction interfaces, and a study has demonstrated that TRAF7’s distinct WD40 domain allows it to interact with certain kinases and receptors to affect stress and inflammatory responses [53]. TRAF7 stimulates and interacts with MEKK3 (*MAP3K3*), a crucial signaling protein in the TNF-induced nuclear factor kappa B (NF-κB) activation pathway [54,55], the activator protein 1 (AP1) and C/EBP homologous protein (CHOP) activation pathways [45], and above all, in the MEKK3-MEK5-ERK5 signaling cascade, which is essential for embryonic vascular integrity and development [48]. Additionally, new and emerging studies draw attention to TRAF7 and its interactions with Robo4, an endothelial-specific receptor that stabilizes the vasculature in pathological angiogenesis by suppressing endothelial hyperpermeability [50,56,57,58,59]. These findings indicate that the TRAF7 protein is involved in diverse biological processes and plays important roles in vascular homeostasis.

### 2.2. Interaction of TRAF7 with Mitogen-Activated Protein Kinases

**Figure 2:**
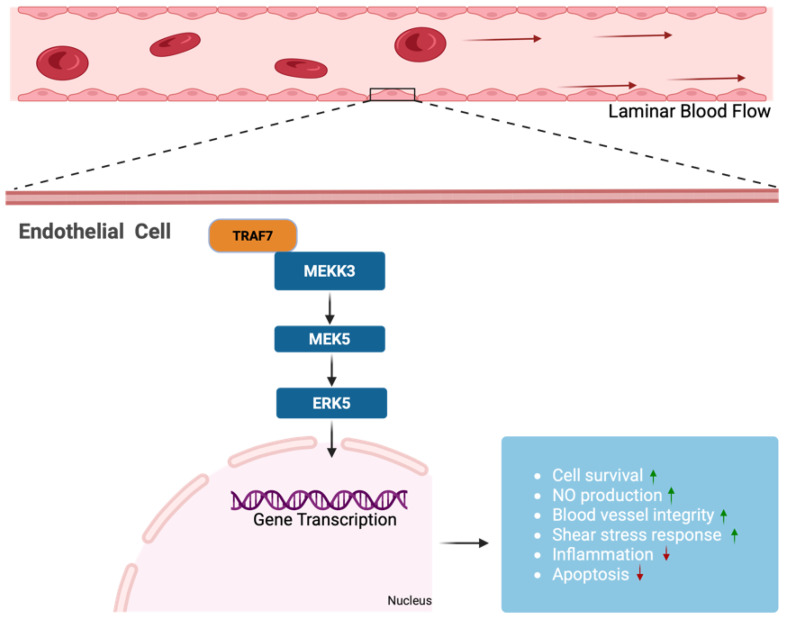
**TRAF7 is a regulator of the shear stress response MEKK3-MEK5-ERK5 signaling pathway**. Blood flow through vessels induces pressure and fluid shear stress response on the endothelial cells that line the luminal surface. The MEKK3-MEK5-ERK5 signaling pathway is activated in a state of laminar blood flow and it exerts protective effects on endothelial cells by promoting anti-inflammatory gene expression, maintaining barrier integrity, and supporting vascular homeostasis. TRAF7 is an upstream regulator of the MEKK3-MEK5-ERK5 signaling pathway.

The interaction between TRAF7 and MAPKs has important implications for mediating cellular responses to a wide range of extracellular stimuli (e.g., growth factors, hormones, stress, and cytokines) and thus regulates cell growth, differentiation, survival, immune response, and neuronal function [60,61,62] as well as endothelial function [48]. Four major MAPK groups have been described in mammals: extracellular signal-regulated kinases (ERK), ERK5, c-Jun N-terminal kinases (JNK), and p38 [61,63]. Each group features a triple sequentially acting kinase module consisting of MEKK3, which phosphorylates and activates a MAPK kinase (MAPKK) that can in turn activate and stimulate the terminal MAPK [61]. TRAF7 specifically interacts with MEKK3 through its unique WD40 repeat domain (Figure 2). This is thought to be functionally significant because co-expression of TRAF7 and MEKK3 results in the activation of major MAPK pathways [45,46]. Xu et al. reported the specific interaction of TRAF7 and MEKK3, and inferred that TRAF7 potentiates MEKK3 kinase activity and, in turn, MEKK3-driven activation of AP1 and CHOP transcription factors through the activation of JNK and p38 MAPK pathways [45]. Functional mapping of protein interactions related to the TNF-α/NF-κB signaling pathway by Bouwmeester et al. identified TRAF7 in association with MEKK3 using tandem affinity purification [46]. Further analysis demonstrated that MEKK3 phosphorylated TRAF7 in a MEKK3-dependent manner, which enabled TRAF7 ubiquitination [46]. Co-expression of TRAF7 and MEKK3 promoted the activation of NF-κB, p38, and JNK pathways, which is in line with results from Xu et al. [45]. Altogether, these findings indicate the crucial role of TRAF7 in regulating the activities of MEKK3 and subsequent activation of the p38 pathway involved in inflammation and the JNK pathway which controls cellular stress and apoptotic response.

MAP kinases, beyond their roles in inflammation and cellular stress responses, have been implicated in the regulation of blood vessel integrity and endothelial function. Knockout studies in mice have identified the MEKK3-MEK5-ERK5 signaling pathway as a critical regulator in maintaining blood vessel integrity. Hayashi et al. demonstrated that genetic deletion of ERK5 in adult mice using the Cre-mediated recombination (ERK*^flox/flox^*) and treatment with polyinosinic–polycytidylic acid resulted in lethality after 2–4 weeks [64]. Further histological analysis revealed heart, brain, and lung hemorrhages, vascular leakage due to increased vascular permeability, and endothelial apoptosis, indicating an impaired structural integrity of the vasculature [64]. Similar phenotypes have been observed in MEKK3 knock-out (KO) mice, in which there was a disruption in the development of blood vessels and embryonic death at around E10.5, thereby suggesting a defect in the function of endothelial cells [65,66,67].

New and emerging studies implicate a role for TRAF7 in the MEKK3-MEK5-ERK5 signaling pathway in endothelial cells. Tsitsikov et al. reported that TRAF7 global KO mouse embryos displayed cerebral hemorrhages and death at E10.5 [48]. Similar phenotypes have also been observed in ERK5, MEKK3, and MEK5 KO mice embryos [65,67,68,69,70]. Tsitsikov et al. also observed discontinuous and less branched blood vessels in TRAF7 KO embryos when compared to wild-type embryos. Further examination of TRAF7 EC KO embryos revealed visible hemorrhage with fragile and damaged blood vessels in the head and back of the trunk. This led to embryonic lethality at E10 as seen in TRAF7 global KO embryos and MEKK3 EC KO embryos. Indeed, they also demonstrated that post-natal TRAF7 EC KO mice had focal brain hemorrhages and blood vessels that were friable and prone to rupture [48]. Similar results were observed by Fisher et al., wherein post-natal loss of MEKK3 in the endothelium was associated with severe hemorrhage in the brain [71]. Altogether, these findings indicate that TRAF7’s engagement with MAPKs, particularly through its interaction with MEKK3, is critical for activating signaling pathways such as the MEKK3-MEK5-ERK5 cascade. This pathway is essential for maintaining endothelial cell integrity, mediating responses to shear stress, and preserving vascular homeostasis [64]. In endothelial cells, these signaling events regulate cellular processes including survival, permeability, and inflammatory responses. Disruptions in these pathways can lead to endothelial dysfunction, which is a key factor in various vascular diseases and cerebrovascular aging (Figure 2) (Table 1) [72,73].

### 2.3. Role of TRAF7 in Endothelial Cell Function and Inflammation Regulation

ECs line the inner surface (intima) of the vasculature in an continuous manner to create a semi-permeable barrier [50,72], and maintaining the integrity of this endothelial cell layer is critical in preventing a range of pathologies since the endothelium is involved in such functions as regulating vascular tone and inflammation and controlling the transport of fluid, proteins, and blood cells between the interstitial and intravascular spaces [79,80]. Endothelial dysfunction has been implicated in the genesis and progression of a variety of pathological conditions such as vascular inflammation, tumor metastasis, and neurodegenerative disorders [81,82,83]. Under healthy and non-inflammatory conditions, endothelial cells adhere to each other tightly via cell–cell junctions made up of vascular endothelial (VE)-cadherins, claudins, occludins, connexins, and endothelial cell selective adhesion molecules [73,83,84]. These proteins work together to maintain blood fluidity, regulate blood flow, control permeability, and quiesce circulating immune cells [73]. However, when infection or tissue injury occurs, endothelial cells undergo changes such as sustained opening of intercellular junctions and formation of gaps between endothelial cells due to dissociation of VE-cadherin-mediated interactions [51]. This is triggered by inflammatory cytokines such as TNF-α and interleukin-1, lipopolysaccharides, pattern recognition receptors, vascular endothelial growth factor (VEGF), and activated immune cells [50,80,81,83]. Exposure of the endothelium to these conditions of increased stress activates the inflammatory NF-κB and AP1 signaling cascades, which upregulate pro-inflammatory responses that lead to increased blood flow and increased vascular leakage of plasma protein. This leads to increased immune cell recruitment at the site of inflammation [73]. Changes in shear stress from laminar to turbulent, low, or oscillating flow have also been reported to cause an altered endothelial phenotype, which includes an increase in monocytes adhesion and upregulation of NF-κB signaling, mediated by nuclear factor erythroid 2–related factor 2 and Krüppel-like factor (KLF) 2 [80].

**Figure 3:**
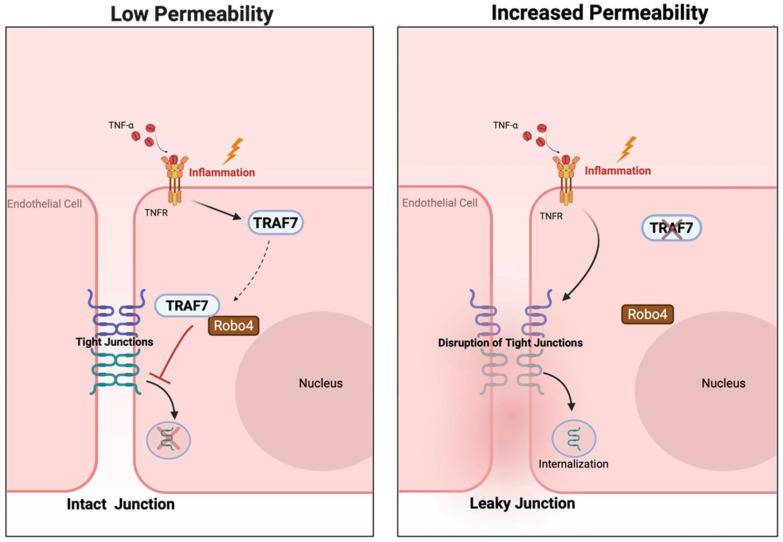
**The TRAF7-Robo4 pathway inhibits TNF-α-mediated endothelial permeability.** Upon inflammatory signaling, TRAF7 translocates from the cytoplasm and interacts with Robo4 in the perinuclear region. The TRAF7-Robo4 complex inhibits TNF-α-mediated disruption and internalization of tight junction proteins to prevent endothelial permeability. A loss of TRAF7 can enhance TNF-α-mediated endothelial permeability.

TRAF7’s role in regulating inflammation in endothelial cells has also been described. Shirakura et al. demonstrated that TRAF7 interacted with Robo4 to suppress endothelial hyperpermeability in inflammation [50]. TRAF7 binds to and interacts with Robo4 through the Robo4 C-terminal domain, which guides TRAF7 localization from the cytoplasm to the perinuclear region (Figure 3) [50]. Shirakura et al. showed that small interfering RNA-mediated knockdown of TRAF7 in human umbilical vein endothelial cells (HUVECs) lowered trans-endothelial electric resistance, a measure of endothelial permeability, after TNF-α stimulation [50]. Conversely, overexpression of TRAF7 in HUVECs using an adenovirus vector suppressed TNF-α-induced hyperpermeability [50]. In a similar manner, knockdown of TRAF7 downregulated the expression of VE-cadherins and increased intercellular gaps at cell junctions in HUVECs, and overexpression of TRAF7 enhanced VE-cadherin localization at cell junctions in HUVECs after TNF-α stimulation [50].

## 3. TRAF7 in the Context of Cerebrovascular Aging

### 3.1. Mechanisms of Cerebrovascular Aging and Associated Cognitive Decline

Cerebrovascular aging encompasses a spectrum of structural and functional changes in the cerebral vasculature, such as compromised cerebral blood flow (CBF) regulation and increased susceptibility to cognitive decline. Vascular remodeling and pathologic changes to the macro- and microvasculature during aging disrupt blood vessel integrity as well [85]. Several age-related cellular mechanisms, such as endothelial dysfunction, are responsible for neurovascular functional impairment. Over time this results in hypoperfusion, BBB breakdown, and neurovascular uncoupling, thereby leading to vascular cognitive decline and dementia [8]. The cerebral endothelium plays a crucial role in maintaining vascular homeostasis by regulating vasodilation, vascular permeability, and inflammatory responses [5,82]. Cerebral endothelial cell dysfunction can eventually lead to dysregulation of CBF and BBB damage, followed by the hyperactivation of the glial and inflammatory environments in the brain [86]. Reduced bioavailability of NO, primarily due to impairment of the eNOS/NO signaling pathway and elevated oxidative stress, leads to impaired vasodilation and vascular stiffness [87]. This dysfunction disrupts the finely tuned regulation of CBF necessary for NVC [86]. Furthermore, cerebral endothelial cells in aged individuals exhibit altered tight junction protein expression, increased transcellular transport, compromising the integrity of the BBB [88]. This results in increased vascular permeability, allowing neurotoxic substances and immune cells to infiltrate the brain parenchyma, thereby triggering neuroinflammation and neuronal injury [89]. Another important mechanism is oxidative stress. This is characterized by the overproduction of reactive oxygen species (ROS), which causes an impairment in antioxidant defense [86]. Mitochondrial dysfunction, nicotinamide adenine dinucleotide phosphate oxidase activation, and a decline in superoxide dismutase activity are major contributors to ROS accumulation in aging vasculature [16]. ROS reacts with NO to generate peroxynitrite (ONOO−), which can easily penetrate the endothelial cell membrane and lead to the macromolecular, lipid, and DNA oxidation associated with vascular aging [90,91,92]. Oxidative stress also promotes vascular remodeling by stimulating the production of extracellular matrix components and contributing to arterial stiffness [93]. These structural changes not only reduce vascular compliance but also hinder the microvasculature’s ability to adapt to the brain’s metabolic demands, exacerbating cognitive deficits associated with aging. Mitochondrial ROS contribution to aging has been reported, and this is exacerbated in conditions of inefficient nutrient oxidation, causing both arterial stiffness, diminished endothelial vasodilation, and increased endothelial apoptosis rate [16,94,95].

Studies show that inflammation plays a role in cerebrovascular aging [96]. Inflammation arises from the sustained activation of innate immune pathways, particularly NF-κB signaling, which drives the expression of pro-inflammatory cytokines such as interleukin-6 (IL-6), interleukin-1β (IL-1β), and TNF-α [97]. This causes endothelial cells to undergo morphological and functional modifications [80], which are usually harmful and contribute to the onset of vascular diseases [11]. Pro-inflammatory cytokines impair endothelial cell function by promoting leukocyte adhesion, disrupting tight junction integrity, inducing oxidative stress, and leading to endothelial apoptosis [98]. This creates a feed-forward loop of inflammation and vascular damage, accelerating cerebrovascular aging and increasing the risk of neurodegenerative diseases such as Alzheimer’s disease. TRAF7 plays regulatory roles in inflammation and oxidative stress. By modulating NF-κB and MAPK signaling pathways, TRAF7 may influence inflammation and oxidative balance within endothelial cells [42]. TRAF7 has also been implicated in apoptotic and survival pathways, which could impact endothelial cell longevity and resilience in the face of aging-related stressors [42].

### 3.2. Impact of Aging on Endothelial Function and BBB Integrity

Aging is accompanied by a progressive decline in endothelial cell function, which has profound consequences for the BBB integrity and overall cerebrovascular health. With advancing age, endothelial cells experience increased oxidative stress, upregulated DNA damage pathways [99,100,101], chronic inflammation, and reduced NO bioavailability, all of which contribute to impaired vasodilation and vascular stiffness [5,10,23,26,102]. These changes compromise the tight junctions that maintain BBB integrity, leading to increased permeability that permits neurotoxic substances and inflammatory cells to infiltrate the brain parenchyma [11]. Recent studies using advanced imaging and single-cell transcriptomic analyses have provided further insights into the molecular alterations in the aging cerebral endothelium. For example, disruptions in endothelial signaling pathways, including those regulated by the MEKK3-MEK5-ERK5 cascade, have been implicated in the reduced capacity of endothelial cells to respond to shear stress and maintain homeostasis [48,64]. This diminished responsiveness contributes to dysregulated CBF and impaired NVC, which are critical for efficient nutrient delivery and waste removal in the brain [103,104]. In addition, age-related upregulation of pro-inflammatory cytokines and oxidative mediators has been linked to endothelial cell senescence, further exacerbating BBB breakdown and facilitating the progression of cerebrovascular diseases [9,105,106,107]. The interplay between endothelial dysfunction and BBB impairment is increasingly recognized as a central mechanism in the pathogenesis of vascular cognitive impairment and dementia. Studies have demonstrated that even subtle increases in BBB permeability can disrupt neuronal homeostasis and trigger neuroinflammatory responses that contribute to cognitive decline [108,109]. Moreover, alterations in the expression of tight junction proteins, such as claudins and occludins, in aged endothelium underscore the vulnerability of the BBB to the cumulative effects of oxidative damage and inflammatory stress [110,111]. Emerging evidence also suggests that interventions aimed at restoring endothelial function [112,113,114], through antioxidant therapy, anti-inflammatory agents, or lifestyle modifications, may hold promise for preserving BBB integrity and mitigating age-related cognitive deficits [115,116]. Taken together, the impact of aging on endothelial function and BBB integrity represents a critical nexus in cerebrovascular aging. A deeper understanding of these processes is essential for developing therapeutic strategies to maintain vascular health and protect brain function in the elderly.

### 3.3. Role of TRAF7 in Modulating Endothelial Responses to Shear Stress and Inflammation

Recent evidence highlights that TRAF7 plays a pivotal role in modulating endothelial responses to both mechanical forces and inflammatory stimuli. Under conditions of shear stress, TRAF7 interacts with MEKK3 to activate the MEKK3-MEK5-ERK5 cascade, a signaling pathway essential for maintaining endothelial integrity, regulating cell alignment, and facilitating adaptive responses to CBF [48,117]. Activation of this cascade supports endothelial cell survival, modulates gene expression, and ensures vascular stability under hemodynamic stress. In parallel, TRAF7 modulates inflammatory responses in endothelial cells by interacting with the receptor Robo4. This interaction is critical for stabilizing endothelial junctions, reducing hyperpermeability, and thereby preserving the integrity of the BBB [50,78]. Experimental evidence also indicates that TRAF7 influences the localization and expression of junctional proteins such as VE-cadherin, which further enhances barrier function and limits the infiltration of inflammatory cells [78,118]. Moreover, TRAF7’s regulation of inflammatory mediators via downstream MAPK signaling pathways has been linked to improved endothelial function in models of vascular injury and stress [119,120]. Collectively, these findings underscore TRAF7’s role as an integrative modulator of endothelial responses, coordinating signals from mechanical forces and inflammatory cues to preserve vascular homeostasis and protect against cerebrovascular dysfunction.

## 4. TRAF7 and Vascular Fragility

Vascular fragility refers to the increased susceptibility of blood vessels to damage and dysfunction. This concept is particularly relevant in the context of aging, where weakened vascular structures contribute to the heightened risk of hemorrhage and ischemic events [16,93,100,121,122,123]. Clinically speaking, the single most profound risk factor for cerebrovascular and cardiovascular disease is advanced age. And, unfortunately, no caliber of vessels is immune to the repercussions of aging [94]. Macrovascular disease, characterized by stiff atherosclerotic plaques in large arteries, causes narrowing of vessels and relative distal hypoperfusion of vital organs. Microvascular pathology has a negative impact on tissue oxygenation, waste export, and nutrient transport. Aging also impacts the blood vessels’ ability to vasodilate in settings of increased demand and relative hypoxia, and vasoconstrict when tissue parenchyma is being hyperperfused. In settings of chronic vascular constriction, such as in persistent hypertensive states seen commonly in aging, the microvasculature can remodel, resulting in a reduction in the number and density of tiny capillaries, a phenomenon known as vascular rarefaction [98,105,124,125,126,127]. Additionally, the general alterations of the secretory phenotype of aged microvascular cells can lead to a wide array of changes in the production of various circulating cytokines, exosomal factors, lipid mediators, and trophic factors that contribute to the age-related alterations seen in the humoral and cellular immune microenvironment of tissues. Moreover, blood vessels can become increasingly leaky in aged individuals. This pathological increase in vascular permeability of the BBB, for example, can lead to the extravascular deposition of misfolded proteins seen in certain tauopathies. On a more macroscopic level, this permeability can result in blood vessel hemorrhages that are the hallmark of vascular fragility [12,75,127,128,129].

There is mounting data that suggests that TRAF7 plays a crucial role in maintaining vascular stability. In the aorta of diabetic rats, TRAF7 inhibits the degradation of Kruppel-like factor 4 (KLF4), which in turn prevents endothelial barrier damage [75,117]. In contrast, TRAF7 causes degradation of KLF4 via N-terminal ubiquitination in a hepatocellular carcinoma cell line, indicating tissue-specific regulation [74]. In another study, the anesthetic agent propofol attenuated the progression of apoptosis and cell injury induced by oxidized low-density lipoprotein in human umbilical vein endothelial cells by upregulating TRAF7 expression [77]. In the setting of pathological angiogenesis induced by VEGF, Robo4 plays an important role in stabilizing the vasculature and limiting unwanted vascular hyperpermeability. One such mechanism is through the interaction of Robo4 with TRAF7 through the C-terminus of Robo4. In this way, the Robo4-TRAF7 complex may serve as an important regulator of inflammation-induced hyperpermeability as previously discussed. Interestingly, when the brains of mice who naturally lived exceptionally long (>34 months) were examined, one of the few genes that was over-expressed was TRAF7 [50,56,57,58,59,118]. While not specified in this report, if the increased expression of TRAF7 translated to a higher prevalence of this protein in vascular endothelial cells, this could suggest that there is a gradient effect, with higher levels of TRAF7 correlating with healthier cerebrovascular aging.

TRAF7 has been implicated in multiple signaling pathways, including the activation of NF-κB and AP-1. It was not until recently that TRAF7 was shown to play a role in a MEKK3-associated protein complex and found to be important in MEKK3-mediated activation of AP-1 [45,54,65,71]. TRAF7 affiliates with many other molecules and pathways as well. Specifically, MEKK3 interacts with the MEK5-ERK5-KLF2 signaling pathway in endothelial cells, which is activated by shear stress and resists apoptosis. While much has been elucidated about the MEKK3-MEK5-ERK5-KLF2 pathway’s essential role in anti-inflammation in endothelial cells, the way in which shear stress affects this remains uncertain [105,110,111,117]. In one study, mouse embryos with a global knockout of TRAF7 began exhibiting signs of growth delay and cerebral hemorrhage at the phase of development that corresponds to the onset of blood flow and the propagation of a functional circulatory system, suggesting that TRAF7 may play a role in blood vessel integrity. In another vivo study, a TRAF7 germline knockout model induced embryonic developmental abnormalities in zebrafish. It is thought that mutations in the coiled-coil (CC) domain of the protein that prevent trimerization could be the cause of the clinical findings [48,76].

## 5. Limitations

Despite the growing interest in TRAF7 and its roles in vascular biology, several important limitations constrain our current understanding, particularly in the context of cerebrovascular aging.

Firstly, most functional insights into TRAF7 come from developmental or non-brain-specific models, such as global or endothelial cell-specific knockout mice and cultured HUVECs (Table 1). While these models are informative, they do not fully replicate the complex and dynamic environment of the aging cerebrovasculature, limiting the translational relevance of the findings. Notably, no studies to date have used aged animal models or brain endothelial-specific TRAF7 knockouts in aged animal models to evaluate age-dependent or neurovascular-specific phenotypes.

Next, many of the studies available focus on TRAF7’s roles in vascular development, inflammation, or tumor biology, with only limited work directly examining its function in neurovascular integrity, BBB regulation, or vascular cognitive impairment—all key hallmarks of cerebrovascular aging. Additionally, there is little known about TRAF7’s role in other key aging pathways (e.g., senescence and oxidative stress). Consequently, a mechanistic understanding of how TRAF7 contributes to age-related microvascular decline remains hypothetical.

Moreover, there is a lack of human data directly linking TRAF7 to cerebrovascular pathologies. While germline mutations in TRAF7 are associated with syndromes affecting multiple organ systems (including the vasculature), there are currently no robust genomic, transcriptomic, or proteomic datasets evaluating TRAF7 expression or function in human brain vasculature during aging.

Finally, TRAF7’s involvement in multiple signaling pathways (e.g., MEKK3-ERK5, NF-κB, Robo4) poses a challenge for therapeutic targeting, as perturbation of TRAF7 could lead to unintended effects in other cellular contexts, such as immune modulation or tumor suppression. The context- and tissue-specific roles of TRAF7 must be carefully delineated before clinical translation can be safely pursued.

Thus, addressing these limitations will require the development of age-specific, cell type-resolved in vivo models, human cerebrovascular datasets, and transcriptomic and proteomic studies.

## 6. Future Directions

Recent studies have provided new insights into the role of TRAF7 in endothelial dysfunction and the contributions to cerebrovascular diseases. For example, research using genetic models has demonstrated that loss or dysregulation of TRAF7 in endothelial cells results in vascular abnormalities, including cerebral hemorrhages and impaired vessel integrity, highlighting its important role in maintaining vascular homeostasis [29,30,31]. Additionally, TRAF7’s modulation of key signaling cascades such as the MEKK3-MEK5-ERK5 pathway is critical for transducing shear stress and inflammatory signals into adaptive cellular responses, further linking its function to cerebrovascular health [29,30,31].

Building on these findings, data suggest that targeting TRAF7 could offer novel therapeutic strategies to mitigate endothelial dysfunction and cerebrovascular aging. Pharmacological approaches, such as the use of antioxidant and anti-inflammatory therapies, may be optimized by combining them with interventions that modulate TRAF7 activity to restore endothelial function and preserve BBB integrity [130,131,132]. Potential TRAF7-specific therapeutics could play a role at a number of consequential key points in the aforementioned pathways. For example, they could ameliorate the interaction between TRAF7 and Robo4/MEKK3 via its unique WD40 repeat domain, improve MEKK3-driven transcription factor activation (e.g., AP1, CHOP), facilitate the relocation of the TRAF7-Robo4 complex to the cell membrane, etc. TRAF7’s role in a number of various signaling pathways, however, could serve as a complicating factor in using small molecules to selectively modulate the protein’s interactions with MEKK3 and Robo4. Gene therapy approaches are also being explored as a means to titrate TRAF7 expression, potentially preventing the cascade of events that lead to endothelial senescence, BBB breakdown, and subsequent cognitive decline [133,134].

Despite these promising advances, several gaps in our understanding remain. Key questions include further clarification of how TRAF7 functions vary across different vascular beds, in different tissues and organs, and under diverse physiological conditions the deciphering of the precise molecular mechanisms by which TRAF7 coordinates responses to shear stress and inflammatory stimuli. TRAF7’s involvement in the MEKK3-MEK4-ERK5 pathway’s role in shear stress response and the Robo4 pathway’s role in preventing endothelial hyperpermeability suggests the possibility of an intersection between these otherwise distinct mechanisms for vascular integrity. Whether a poor shear stress defense facilitates a pro-inflammatory response leading to hyperpermeability or whether the pathways are cooperative but not directly sequential should be a focus of further scientific inquiry. Additionally, little is known about any potential interaction or crosstalk between TRAF7 and its other TRAF counterparts.

Also critical is how preclinical insights could be effectively translated into clinical interventions for vascular diseases and age-related cognitive disorders. Addressing these questions will require comprehensive longitudinal studies, advanced in vivo models, and detailed molecular investigations. A deeper understanding of TRAF7’s multifaceted role in endothelial biology is essential not only for validating it as a therapeutic target but also for developing interventions that can preserve cerebrovascular integrity and cognitive function in the aging population.

## 7. Conclusions

This review highlights the emerging role of TRAF7 as a critical regulator of cerebrovascular health. TRAF7’s unique structural features, marked by the absence of a traditional TRAF-C domain and the presence of seven WD40 repeats, enable it to interact with key signaling molecules, notably MEKK3, thereby modulating pathways such as the MEKK3-MEK5-ERK5 cascade. These interactions are vital for maintaining endothelial cell function, regulating vascular permeability, and preserving the integrity of the BBB. The evidence reviewed indicates that TRAF7 not only influences cellular responses to mechanical forces and inflammatory stimuli but also plays a significant role in preventing endothelial dysfunction and, consequently, cerebrovascular aging. The implications of these findings extend beyond basic biological insights. Understanding TRAF7’s function opens avenues for novel therapeutic strategies aimed at mitigating age-related vascular decline and cognitive impairment. Potential interventions, including pharmacological modulation and gene therapy approaches targeting TRAF7, could restore endothelial homeostasis and protect against the progression of cerebrovascular diseases. These prospects underscore the broader significance of TRAF7 in advancing vascular medicine and developing treatments to preserve cognitive function in the aging population. TRAF7 may yet emerge as a promising target in the quest to understand and combat cerebrovascular aging. Its central role in regulating endothelial function and vascular integrity makes it a key focus for future investigations and therapeutic development. Further research is essential to fully elucidate its mechanisms and translate these findings into the clinical arena, ultimately enhancing vascular health and preventing cognitive decline.

## Figures and Tables

**Table 1:** Comparison of TRAF7 models.

Disorder/Model(s)	Findings	Limitations/Clinical Relevance	Reference
No disorder Human tissues HEK293 cells	Human TRAF7 mRNA detected in (highest to lowest) skeletal muscle, heart, brain, kidney, placenta, spleen, colon, small intestine, brain, thymus, lung, leukocytes TRAF7 binds MEKK3 TRAF7 interaction with AP1 and CHOP	Not a study of a disease process, only characterization of some TRAF7 interactions. Cell culture results may not reflect tissue-specific TRAF7 interactions.	Xu, L-G et al., 2004 [45]
No disorder A529 cells HeLa cells HEK293 cells	TRAF7 activates NF-κB promoter. TRAF7 enhances TRAF6 activation of NF-κB promoter. TRAF7 participates in phosphorylation of IκBα and p38 and induction of TNF-α, IL-1β, and IL-8. CYLD inhibits TRAF6 and TRAF7 ubiquitination.	Not a study of a disease process, only characterization of some TRAF7 interactions. Cell culture results may not reflect tissue-specific TRAF7 interactions.	Yoshida H et al., 2005 [55]
No disorder HEK293 cells HeLa cells Mouse embryonic fibroblasts	TRAF7 facilitates TNF-α activation of AP1 promoter TRAF7 facilitates TNF-α-mediated phosphorylation of JNK. TRAF7 ubiquitinates c-FLIPL, but not JNK.	Not a study of a disease process, only characterization of some TRAF7 interactions. Cell culture results may not reflect tissue-specific TRAF7 interactions.	Scudiero I et al., 2012 [47]
Hepatocellular carcinoma (HCC) PLC5 cells HepG2 cells MHCC97H cells MHCC97L cells HEK293 cells Patient tissue samples–tumor and healthy tissue	Increased TRAF7 mRNA in HCC compared to normal tissues HCC with higher TRAF7 expression had less disease-free survival and less overall survival. In HCC cells: TRAF7 expression negatively correlated with KLF4 expression. KLF4 expression was reduced by TRAF7 ubiquitination of KLF4. TNF-α decreased TRAF7 expression, which inhibited KLF4 degradation. IL-6 increased TRAF7 expression, which facilitated KLF4 degradation. TRAF7 did not affect apoptosis. TRAF7 promoted metastasis. In normal cells: TRAF7 increased KLF4 ubiquitination. TRAF7 binds KLF4 via the RING finger and coiled-coil domains. The N-terminal of KLF4 (1–60 amino acids) is required for TRAF7 ubiquitination.	Mechanism of KLF4 regulation by TRAF7 in HCC may not apply to other cancers. The interaction in normal cell lines was due to overexpression or knockdown of expression; the TRAF7-KLF4 interaction may not be physiologically relevant for oncogenesis.	He H et al., 2020 [74]
Diabetes (Type 1 or 2) db/db or wild type mice Streptozotocin (STZ) treated Wistar rats Mouse aortic endothelial cells (MAECs)	Db/db mice (aorta) had higher TRAF7 expression, which was reversed by NaSH. Wistar rats treated with STZ and fed a high-fat diet had increased TRAF7 expression (aorta) that was inhibited by NaSH treatment. In MAECs, treatment with high glucose and palmitate (a model of diabetes) increased TRAF7 expression and was reversed with NaSH. TRAF7 ubiquitination of KLF4 was decreased by NASH. In the diabetes cell culture model, TRAF7 decreased Nrf2, VE-cadherin, β-catenin, and occludin expression. H_2_S sulfhydration of TRAF7 inhibits modulation of KLF4.	Animal models of diabetes have limited translation to chronic diabetes. The majority of the mechanism was determined using MAECs, which are specialized and may not represent the physiology of other endothelial cells. Uncertain if the MAEC diabetic model is relevant to chronic diabetes.	Li Q et al., 2024 [75]
No disorder Floxed (fl) TRAF7 mice TRAF7^fl/fl^:E2a-Cre mice TRAF7^fl/fl^:Tie2-Cre TRAF7^fl/fl^:Cdh5 (PAC)-CreERT2 Human umbilical vein endothelial cells (HUVECs) HEK293 cells	Global TRAF7 deletion was embryonic lethal due to developmental heart defects. Endothelial TRAF7 deletion was embryonic lethal. Postnatal endothelial TRAF7 deletion caused lethal brain hemorrhage. TRAF7 bound MEKK3, MEKK2, MEK5, and SCRIB. MEKK3 and MEK5 bound to the C-terminal WD40 domain of TRAF7. MEK5 and SCRIB bound to the RING and zinc finger domains of TRAF7. Phosphorylation of ERK5 increased in HUVECs exposed to shear stress mediated by TRAF7, SCRIB, and MEKK3. Expression of KLF2 and KLF4 was also increased in HUVECs exposed to shear stress.	Not a study of a disease process, only characterization of some TRAF7 interactions. The lethal TRAF7 deletion in the mouse model does not reproduce the clinical TRAF7 syndrome, which causes developmental delay and other abnormalities. Cell culture results may not reflect tissue-specific TRAF7 interactions. The shear stress cell culture model may have limited translatability to clinical vascular disorders.	Tsitsikov E et al., 2023 [48]
No disorder Wild-type or red fluorescent protein-expressing zebrafish	TRAF7 expression in zebrafish can be monitored throughout development, with the highest expression in the brain. Knockdown of TRAF7 causes abnormal development in zebrafish. The coiled-coil domain of TRAF7 is necessary for zebrafish development.	Not a study of a disease process, only characterization of some TRAF7 interactions. Zebrafish are commonly used to study developmental biology since they are transparent and can be used in large numbers. Zebrafish biology may not accurately model human biology/disease.	Song X et al., 2024 [76]
Atherosclerosis (AS) Serum samples from healthy volunteers and AS patients HUVEC model of AS induced by oxidized low-density lipoprotein (ox-LDL)	In HUVECs, ox-LDL caused dose and time-dependent decreases in cell viability and increases in apoptosis; propofol treatment reversed the ox-LDL effects. Circular RNA, Circ_0003645, expression was increased in AS serum and ox-LDL-treated HUVECs. Propofol treatment reduced Circ_0003645 in ox-LDL-treated HUVECs. miR-149-3p was decreased in AS serum and HUVECs treated with ox-LDL, due to Circ_0003645, and restored with propofol treatment. TRAF7 increased in AS serum and ox-LDL-treated HUVECs and decreased by propofol treatment. miR-149-3p decreased TRAF7 expression, and the effect was reversed by Circ_0003645-induced decrease in miR-149-3p.	While a mechanism for the modulation of TRAF7 in a HUVEC model of AS was developed, this same model was not evaluated in cells from AS patients. The ox-LDL model of AS in HUVEC may not accurately model chronic disease.	Chen M et al., 2023 [77]
Inflammation-Induced Endothelial Hyperpermeability Robo4 knockout mice HUVECs HEK293 cells COS-7 cells	In Robo4^-/-^ mice, lipopolysaccharide (LPS)-induced permeability in the heart, lung, and small intestine. Robo4 inhibits TNF-α-induced endothelial hyperpermeability. Robo4 increases VE-cadherin localization to endothelial cell junctions. TRAF7 interacts with the C-terminal of Robo4. TRAF7 is necessary for Robo4 inhibition of endothelial hyperpermeability.	Cell culture results may not reflect tissue-specific TRAF7 interactions. The importance of Robo4 signaling in inflammation-induced hyperpermeability in clinical disorders is unknown.	Shirakura K et al., 2019 [50]
No disorder HEK293 cells	Analysis of the TNF-α-NF-κB signaling pathway. TRAF7 was identified as reducing NF-κB activation. MEKK3 phosphorylates and ubiquitinates TRAF7. TRAF7 WD40 domain interacts with MEKK3. TRAF7 coiled-coil domain is necessary for TRAF7 homodimerization. TRAF7-MEKK3 interaction activates NF-κB, JNK, p38.	Cell culture results may not reflect tissue-specific TRAF7 interactions. Modulation of inflammatory signaling may be relevant for treating chronic inflammatory diseases.	Bouwmeester T et al., 2004 [46]
TRAF7 Syndrome Genetic testing of patients Skin biopsy fibroblast cell culture	45 patients with TRAF7 germline variants were identified. All variants were within the WD40 region of TRAF7. mRNA expression of selected differentially expressed genes were verified.	Many of the germline variants were unique, indicating there is not a single key variant to target for treatment of the syndrome.	Castilla-Vallmanya L. et al., 2020 [49]
Inflammation-Induced Endothelial Hyperpermeability HUVECs HEK293 cells COS-7 cells Robo4 knockout mice –collagen-induced arthritis model	Robo4 and TRAF7 interact to ubiquitinate IQGAP1 to suppress RAC1. RAC1 induces PTGS2 expression, which leads to endothelial hyperpermeability via JNK-AP1 signaling.	Cell culture results may not reflect tissue-specific TRAF7 interactions. The importance of Robo4 signaling in inflammation-induced hyperpermeability in clinical disorders is unknown.	Tanaka M et al., 2024 [78]
